# Employment among people with schizophrenia or bipolar disorder: A population‐based study using nationwide registers

**DOI:** 10.1111/acps.13254

**Published:** 2020-11-24

**Authors:** Minna Holm, Heidi Taipale, Antti Tanskanen, Jari Tiihonen, Ellenor Mitterdorfer‐Rutz

**Affiliations:** ^1^ Mental Health Unit Finnish Institute for Health and Welfare Helsinki Finland; ^2^ Department of Clinical Neuroscience Karolinska Institutet Stockholm Sweden; ^3^ School of Pharmacy University of Eastern Finland Kuopio Finland; ^4^ Department of Forensic Psychiatry Niuvanniemi Hospital University of Eastern Finland Kuopio Finland; ^5^ The Impact Assessment Unit National Institute for Health and Welfare Helsinki Finland

**Keywords:** bipolar disorder, employment, income, pensions, schizophrenia

## Abstract

**Objective:**

To assess the employment rate and the related background factors among people with schizophrenia or bipolar disorder.

**Methods:**

We identified all people in Sweden aged 18–64 years diagnosed with schizophrenia or bipolar disorder in nationwide registers in the years 2006–2013. The identified individuals were grouped by main activity or source of income. The association between background factors and employment was analyzed with generalized estimating equations (GEE).

**Results:**

Three years before the first psychosis or bipolar disorder diagnosis, 24% of the individuals with schizophrenia and 45% of the individuals with bipolar disorder were employed. However, the employment rate dropped around the time of the first diagnosis. Five years later, 10% of the individuals with schizophrenia and 34% of the individuals with bipolar disorder were employed. The most important factors associated with employment after diagnosis were a high level of education, older age at the first registered diagnosis, no substance use disorder, and a low number of previous hospitalizations. Marriage or cohabiting, higher level of education, and higher age at the first diagnosis were associated with an increased employment rate especially among people with schizophrenia, and substance use was associated with a lower employment rate, especially among people with bipolar disorder. Men with bipolar disorder had a higher employment rate than women.

**Conclusion:**

The employment rate is low among people with schizophrenia and higher among people with bipolar disorder. The association of background characteristics with employment was mostly in the same direction both in schizophrenia and in bipolar disorder.

**Significant outcomes**

- Among people with schizophrenia, employment was rare, and over 80% were on a disability pension.
- Among people with bipolar disorder, employment was more common than in schizophrenia; however, the majority were on a disability pension or on long‐term sick leave as well.
- Employment rate varied considerably within the diagnosis groups in relation to demographic and illness characteristics.

**Limitations**

- Only information that could be obtained from the registers was available, and therefore, we had no knowledge of symptom severity.
- Differentiation between Bipolar I and Bipolar II disorders was not possible.

## 1 INTRODUCTION

Mental disorders are among the most common causes of disability in working‐age people in the world.^1^, ^2^ Bipolar disorder and schizophrenia are among the most severe mental disorders due to the significant decline in everyday functioning, such as work participation, social relationships, and ability to live independently, and the drop occurs already early in life.^3^, ^4^ People with bipolar disorder have on average a better functional outcome than people with schizophrenia^5^; however, many people with bipolar disorder also experience a severe decline in vocational and social functioning, even when their mood is euthymic.^6^

The employment rate among people with bipolar disorder has been estimated to be 40%–60%^7^ and among people with schizophrenia 10%–30%.^8^, ^9^, ^10^ However, only a few studies using large, population‐representative samples assessing the employment or disability pension rate in both schizophrenia and bipolar disorder have been published. A Swedish register‐based study reported that 40% of people with bipolar disorder were employed, but among the employed, sickness absence was common.^11^ In a Finnish register‐based study, 14% of persons with schizophrenia and 43% of persons with bipolar disorder were employed.^12^ In another population‐representative study, 80% of all people with schizophrenia and 38% of people with an affective psychotic disorder were pensioned.^8^

In previous studies, being unemployed has been related to lower cognitive and social functioning, higher levels of negative and depressive symptoms as well as lower levels of education in individuals with schizophrenia or bipolar disorder.^5^, ^13^, ^14^, ^15^, ^16^, ^17^ Employment has, besides salary, been related to higher self‐esteem and higher quality of life^14^, ^18^ and may improve clinical outcomes.^19^, ^20^, ^21^ Waghorn et al^22^ noticed that the correlates of employment differed among people with schizophrenia and bipolar disorder with age, illicit drug use and insight, predicting employment among people with bipolar disorder and education and self‐care performance in schizophrenia, but the number of currently employed were only 42 out of 156 in the bipolar group and 61 out of 385 in the schizophrenia group. In the few previous studies with large, population‐based samples, the assessment of background characteristics of employment has been scarce, and these studies have mostly concentrated on either schizophrenia or bipolar disorder; therefore, a comparison of these disorders has not been possible.

However, it is not just personal factors that matter when patients with schizophrenia or bipolar disorder manage to establish themselves on the labor market—structural characteristics are also important. It is therefore necessary to interpret the results of studies on employment rates in these patient groups in light of prevailing social security and labor market policies in the country where the study is conducted. Sweden provides a generous social security system and a variety of labor market policy programs. Labor market policy measures include not only financial support for the unemployed in the form of unemployment benefits but also employment services, labor market training, and employment support. Moreover, work adaptations for individuals with functional impairment are easily accessible, and different forms of vocational rehabilitation such as supported employment are available. In the case of work incapacity, economic support related to sickness absence and disability pension can be granted.

### 1.1 Aims of the study

In order to close the current knowledge gap, the aim of the present study was to assess the employment rate and the related background factors among people with schizophrenia or bipolar disorder in the Swedish population.

## 2 METHODS

### 2.1 Study population

The study population was based on all Swedish residents aged 16–64 years in the year 2006. The patient cohorts were selected on the basis of diagnoses in the National Patient Register and Micro‐Data for Analysis of the Social Insurance System (MiDAS) register (containing data on disability pension and sickness absence). The National Patient Register contains all inpatient care since 1987 and specialized outpatient care since 2001 in Sweden. The register has been evaluated to be comprehensive and of good quality.^23^ However, primary health care is not covered by the National Patient Register. All Swedish residents have a unique personal identification number, which enables linking different registers.

Two cohorts were drawn from the registers: a schizophrenia cohort and a bipolar cohort. From the prevalent cohorts, we identified incident samples in order to be able to follow trajectories before and after the onset of the diagnosis. For the schizophrenia cohort, all individuals who were treated due to schizophrenia (International Classification of Diseases 10th Revision, ICD‐10 code F20 as the main diagnosis) in the years 2006–2013 were selected.^24^ For the *incident* schizophrenia sample, we identified all individuals with their first diagnosis of schizophrenia in 2006–2013 and without a previous main or contributory diagnosis of schizophrenia spectrum disorders (F20–29 in ICD‐10 or 295 in ICD‐9) before July 1, 2006, or antipsychotic use between July 1, 2005, and July 1, 2006, according to the Prescribed Drug Register.

For the bipolar cohort, we selected all individuals who had received the main diagnosis of a manic episode or bipolar disorder (ICD‐10‐codes F30‐F31) 2006–2013 in the registers used. The *incident* sample was formed from individuals who had not received bipolar disorder diagnosis during the years 2001–2005 and had their first diagnosis in the years 2006–2013. In addition, people having schizophrenia spectrum disorder (F20–F29) before a bipolar diagnosis were excluded.

### 2.2 Ethics approval

The study project has been approved by the Regional Ethics Board of Stockholm (2007/762‐31). Because the present study is entirely register‐based, no informed consent was required.

### 2.3 Study variables

Information regarding the main activity and source of income was received from the Longitudinal Integration Database for Health Insurance and Labour Market Studies (LISA) register held by Statistics Sweden. The LISA register follow‐up started from the year 2003 for the incident sample and from the year 2006 for the prevalent sample and lasted until the year 2013, 65 years of age, emigration, or death. Employment was analyzed in the adult population (≥18 years). The patient groups were compared to the whole adult population (18–64 years old) in Sweden, which was defined as people living in Sweden on December 31 during each year of the study.^25^

People were classified into mutually exclusive groups based on their main activity or yearly income.^26^ The categorization of employment was based on the Statistics Sweden classification.^25^ The Statistics Sweden categorized people as employed when they received income from work above a certain limit, which varied between professions and years. The limit approximates whether one has been working at least 1 h per week. For people who were unemployed, on parental leave, on sick leave, on a disability pension (measured as gross days) or old‐age pension over half of the year, the respective category was defined as their main activity of the year. People who received more than half of their disposable income from student benefits or social assistance were categorized as such. The remaining individuals were categorized as “other.”

All people with permanently impaired work capacity in Sweden are eligible for a disability pension. In 2008, the eligibility requirements were tightened, and the possibility for a time‐limited benefit was removed for those over 30 years of age. Sickness absence benefits are paid from the Swedish Social Insurance Agency after 14 days of sickness absence for employees or from the second day of absence for the unemployed. In 2008, the requirements for sickness absence benefits were also tightened, and people are required to have assessments at fixed points. People actively looking for work are entitled to unemployment benefits.

Sociodemographic factors, including age, gender, education, marital status (married or cohabiting vs. other), immigration status, and municipality employment rate, were collected from the LISA register (Table S1). Education was categorized into three categories: low (compulsory school, 9 years of education or less), middle (upper secondary education, 10–12 years of education), and high (college or university). Immigration status was defined as whether the person was born in Sweden. Municipality employment rate was defined as the percentage of people in each municipality whose main activity of the year was defined as employed. The age at first diagnosis in the registers (categorized as: <25, 25–34, and >35) was defined based on the date of first diagnosis of F20–F29 or F30–F31 as the main diagnosis in the National Patient Register or in the MiDAS register. Substance use disorders (used as a binary variable, yes/no) were defined as register diagnosis of ICD‐10 codes F10–F19 since the year 2001 and until the year in question (2006–2013). A number of psychiatric hospitalizations were defined as hospitalizations with the main diagnosis of F01–F99 starting from 5 years before the beginning of the follow‐up (from the year 2001) and until the year in question. Number of hospitalizations were categorized as: no hospital care, 1–2 hospitalizations, 3–5 hospitalizations, and more than 6 hospitalizations.

### 2.4 Statistical analysis

People were categorized according to their main activity each year. First, the categories were plotted to examine how common the main activities were during the years 2006–2013 among people with schizophrenia and among people with bipolar disorder, from the first whole year after the first register diagnosis, as well as in the whole Swedish population. In addition, in the incident sample, the categories were plotted to show the main activities between 3 years before and 5 years after the first registered diagnosis in the years 2006–2013.

The association between background characteristics and employment status (defined as the main activity of the year and categorized as employed/not employed) was analyzed with the generalized estimating equations (GEE) model in R version 3.4.1 with the gee package.^27^ The GEE model is used for estimating population‐averaged estimates of parameters when the data are longitudinal. The prevalent schizophrenia and bipolar cohorts were included in the GEE model. The independent variables used were diagnosis (schizophrenia/reference category: bipolar disorder), age (as a continuous variable), gender, educational level, marital status, immigration status, municipality employment rate (as a continuous variable), calendar year, age of first diagnosis in the registers (in the three categories), substance abuse diagnosis in the registers, and number of psychiatric hospitalizations by the year in question. First, interactions between the diagnosis and all other independent variables were included in the GEE model. Next, non‐significant interactions were removed from the analysis (*p* > 0.05). The results were reported as odds ratios (OR) with 95% confidence intervals (CI). The interaction term was reported as the ratio of odds ratios (ROR).

The RORs can be interpreted as follows: if the interaction term (e.g. gender × diagnosis group) is non‐significant, the OR can be interpreted as a measure of the association between the independent variable (e.g. gender) and employment status, regardless of diagnostic group. However, if the interaction term is statistically significant, the ORs are different in the diagnosis groups. Then, the OR of the variable (e.g. gender) can be interpreted as a measure of the association between the variable (e.g. gender) and employment in the bipolar group, whereas in the schizophrenia group, the OR of the variable can be calculated by multiplying the ROR by the OR of the variable. When the ROR is above 1, the OR for an independent variable in the schizophrenia group is higher than in the bipolar group, and when the ROR is below 1, the OR in the schizophrenia group is lower than in the bipolar group.

## 3 RESULTS

### 3.1 Demographic and illness characteristics of people with schizophrenia or bipolar disorder

In the schizophrenia cohort, over half were men, whereas in the bipolar disorder cohort, over half were women (Table 1). People in the general population belonged to the youngest age group (18–25 years, 15%) more often than people with schizophrenia (1%) or bipolar disorder (4%). Of the individuals with bipolar disorder, a quarter had high education, but 16% had only basic education. The individuals with schizophrenia had lower education than the individuals with bipolar disorder or the general population. However, even 12% of the individuals with schizophrenia had high education. People in the general population were more often married or cohabiting (56%) than people with schizophrenia (12%) or bipolar disorder (35%). The number of individuals who had received bipolar disorder diagnosis in the registers increased from 13 081 in 2006 to 35 119 in 2013, whereas the number of the individuals with schizophrenia diagnosis decreased a little (year 2006 = 23 953; year 2013 = 19 881).

**Table 1:** Sociodemographic and illness characteristics in the year 2013 (end of follow‐up) in people with schizophrenia and bipolar disorder as well as in the general population in Sweden

	Schizophrenia		Bipolar disorder		General population	
	*n*	%	*n*	%	*n*	%
*n* in the whole group	19881		35119		5713549	
*n* in the incident group	2781	14	25188	72		
Age (years)^a^						
18–24	253	1	1498	4	865743	15
25–34	2446	12	8304	24	1128240	20
35–44	4057	20	8323	24	1197948	21
45–54	6636	33	8815	25	1260255	22
55–64	6489	33	8179	23	1261363	22
Gender^a^						
Women	7490	38	22082	63	2815947	49
Men	12391	62	13037	37	2897602	51
Education^a^						
Low	6349	32	5614	16	834805	15
Middle	10882	56	19572	56	3118596	55
High	2339	12	9784	28	1704238	30
Marital status^a^						
Married or cohabiting	2395	12	12122	35	3206505	56
Other	17486	88	22997	65	2507044	44
Immigration status^a^						
Born in Sweden	15386	77	30998	88	4769573	83
Born outside Sweden	4495	23	4121	12	943976	17
Municipality employment rate^a^						
Lowest 25% of the municipalities	1735	9	2036	6		
Middle	9769	49	17623	50		
Highest 25%	8377	42	15460	44		
Age of first diagnosis^b^						
<25	3364	17	4634	13		
25–34	6429	32	8905	25		
>35	10088	51	21580	61		
Substance use disorder diagnosis^c^						
No	15833	80	31721	90		
Yes	4048	20	3398	10		
Psychiatric hospitalizations^d^						
No hospitalizations	12967	65	25963	74		
1–2 hospitalizations	3829	19	5778	16		
3–5 hospitalizations	2092	11	2418	7		
>6 hospitalizations	993	5	960	3		

^a^ In the Longitudinal Integration Database for Health Insurance and Labour Market Studies (LISA) register for 2013.

^b^ In the National Patient Register or in the Micro‐Data for Analysis of the Social Insurance System (MiDAS) register.

^c^ International Classification of Diseases version 10 (ICD‐10) codes F10–F19 in the National Patient Register or in the MiDAS register in the years 2001–2013.

^d^ Hospitalizations with ICD‐10 codes F01–F99 in the years 2001–2013.

### 3.2 What do people do from year 2006 to year 2013?

**FIGURE 1:**
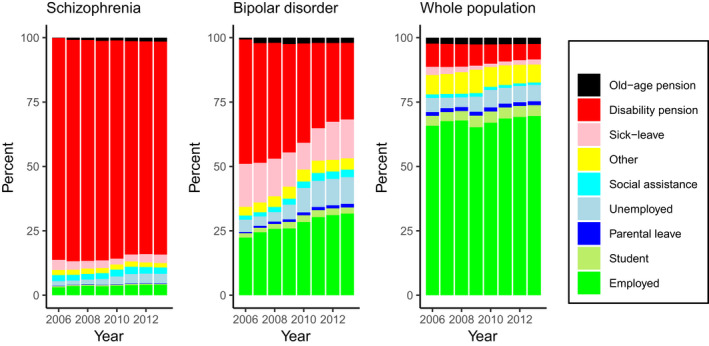
The main activity of people with schizophrenia and with bipolar disorder and in the whole Swedish population during the years 2006–2013

Among people with schizophrenia, the most common category of main activity was disability pension, which slightly decreased from 86% in 2006 to 83% in 2013 (Figure 1). Between 3% and 4% of individuals with schizophrenia were classified as employed, and approximately the same percentage were classified as being on long‐term sick leave. Students or those on parental leave were the smallest categories, with <1% classified as such.

Among people with a bipolar disorder, the proportion of those whose main activity was employed increased from 22% to 32% between the years 2006 and 2013, and the proportion of people on a disability pension declined from 48% to 30%. Long‐term sick leave was the third biggest group, varying between 10% and 17%. Parental leave was the smallest group—approximately 1%.

### 3.3 Different definitions of employment

When employment was defined as receiving a salary that is over the first quartile of the general population, 6% of the individuals with a schizophrenia diagnosis were classified as employed. In bipolar disorder, the proportion rose from 37% in the year 2006 to 39% in the year 2013. Defining employment as working at least 1 h in a week, 13% of the individuals with schizophrenia were employed in the year 2006, decreasing to 11% by the year 2013, and for individuals with a bipolar disorder, 48–54% were classified as employed between the years 2006 and 2013.

### 3.4 The association of demographic factors and clinical factors with employment status

In GEE analyses controlling for sociodemographic and clinical factors, the individuals with bipolar diagnosis had a higher employment rate than the individuals with schizophrenia, but the diagnosis had many statistically significant interactions with covariates (Table 2). Of the clinical factors, a first registered diagnosis at an older age was associated with a higher probability of working than for those younger than 25. The association of age of first registered diagnosis was stronger in the individuals with a schizophrenia diagnosis than in the individuals with a bipolar diagnosis. A registered diagnosis of substance use disorder and a high number of psychiatric hospitalizations was associated with a decreased probability of working, especially among people with bipolar disorder.

**Table 2:** The association of demographic and clinical factors with employment in people with schizophrenia or bipolar disorder and in the general population. GEE analysis was performed in people with schizophrenia or bipolar disorder (beginning in the first whole year after the first register diagnosis) and included the years 2006–2013

	Employment rate in year 2013 (%)			Main effect in GEE analysis		Interaction with diagnosis	
	Schizophrenia	Bipolar disorder	General population	OR^c^	95% CI	ROR^c^	95% CI
Schizophrenia diagnosis (ref. bipolar)	4.1	32.3		0.52	0.39–0.70		
Age^a^, ^d^							
18–24	7.1	32.4	49.7	0.96	0.95–0.96	0.95	0.94–0.95
25–34	6.0	38.2	71.5				
35–44	6.4	37.9	80.3				
45–54	3.9	30.9	78.8				
55–64	2.1	22.0	62.0				
Gender^a^							
Women	3.8	30.5	67.3	0.62	0.60–0.64	1.31	1.15–1.48
Men	4.4	35.2	71.8	1.00			
Education^a^							
Low	1.6	17.5	45.6	1.00			
Middle	4.0	29.8	71.2	1.94	1.82–2.07	1.14	0.94–1.37
High	12.2	46.1	80.0	3.89	3.63–4.17	1.26	1.01–1.55
Marital status^a^							
Married or cohabiting	10.0	39.4	73.8	1.14	1.10–1.17	1.27	1.13 – 1.43
Other	3.3	28.5	64.1	1.00			
Immigration status^a^							
Born in Sweden	4.3	33.1	72.4	1.00			
Born outside Sweden	3.7	26.3	55.2	0.71	0.67–0.75		
Municipality employment rate^a^, ^d^				21.2	14.8–30.4		
Lowest 25% of municipalities	3.3	27.1					
Middle	3.8	29.1					
Highest 25%	4.7	36.6					
Time in years^d^				1.03	1.03–1.04		
Age of first diagnosis^b^							
<25	3.6	34.5		1.00			
25–34	4.3	38.7		1.50	1.41–1.60	1.60	1.31–1.96
>35	4.2	29.2		2.02	1.85–2.21	4.01	3.22–5.21
Substance use disorder diagnosis^b^							
No	4.7	34.2		1.00			
Yes	2.0	14.0		0.49	0.45–0.53	1.22	1.01–1.47
Psychiatric hospitalizations^b^							
No hospital care	5.0	37.1		1.00			
1–2 hospitalizations	3.4	23.1		0.68	0.63–0.70	1.08	1.01–1.15
3–5 hospitalizations	2.0	12.9		0.48	0.46–0.50	1.14	1.03–1.26
>6 hospitalizations	0.3	5.9		0.32	0.29–0.36	1.05	0.85–1.28

^a^ In the Longitudinal Integration Database for Health Insurance and Labour Market Studies (LISA) register.

^b^ In the National Patient Register or in the Micro‐Data for Analysis of the Social Insurance System (MiDAS) register.

^c^ OR = odds ratio of the variable. ROR = ratio of odds ratios. If the interaction term was statistically significant, the odds ratios are different in the diagnosis groups. Then, the OR in the main effects column can be interpreted as the OR in the bipolar group, and the OR of the variable in the schizophrenia group can be calculated by multiplying the ROR by the OR of the variable (e.g., the OR in the schizophrenia group for gender is 0.62*1.31 = 0.81).

^d^ Continuous variables in the GEE model.

Of the sociodemographic factors, the sex difference was small in those with schizophrenia, whereas men with bipolar disorder had a higher employment rate than women with the disorder. Persons who were young, had higher education, and were married were more likely to work, especially among those with schizophrenia. In addition, a higher employment rate was associated with being born in Sweden.

Of the societal factors, a higher employment rate in the municipality was associated with a higher employment rate in the individuals with schizophrenia and bipolar disorder. The employment rate increased during the years 2006–2013.

**FIGURE 2:**
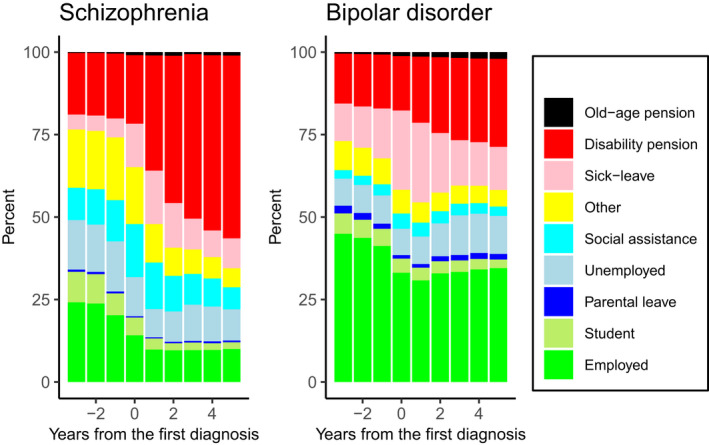
Employment rate between 3 years before and 5 years after the first register diagnosis for the incident samples with the first register diagnosis during the years 2006–2013

Even 24% of the individuals with schizophrenia and 45% of the individuals with bipolar disorder were employed 3 years before the first diagnosis in the registers (Figure 2). However, around the time of the first diagnosis, the employment rate dropped. Five years after the illness onset, 10% of the individuals with schizophrenia and 34% of the individuals with bipolar disorder were employed.

## 4 DISCUSSION

The present nationwide register‐based study investigated work participation in people with schizophrenia or bipolar disorder in Sweden between the years 2006 and 2013. The results suggested that the employment rate among people with bipolar disorder varied between 22% and 32%. Nevertheless, most individuals with bipolar disorder were on a disability pension, long‐term sick leave, or unemployed. Among people with schizophrenia, only 3%–4% were working and over 80% were on a disability pension.

Three years before the first psychosis diagnosis, 24% of the individuals with schizophrenia and 45% of the individuals with bipolar disorder were working. The employment rate among people with schizophrenia or bipolar disorder dropped within a few years before the illness, which is in accordance with previous studies.^12^, ^28^ Among people with bipolar disorder, the employment rate was the lowest right after the illness onset and started to rise a little 2 years after the onset of the illness. The rise may reflect the recovering from the illness, which is in line with the results of Reed et al.^29^ In contrast, among people with schizophrenia, the proportion of the employed was relatively stable after the diagnosis. However, also among people with bipolar disorder, the most obvious change after the diagnosis was the increase in the proportion of people on a disability pension.

Both among people with schizophrenia and with bipolar disorder, the most important variables predicting a higher probability of employment were a high level of education and a low number of previous hospitalizations, in accordance with earlier results.^10^, ^14^, ^17^ On the other hand, the individuals who were not married or cohabiting were older or had a substance use disorder diagnosis were least likely to be employed, in accordance with earlier results.^10^, ^14^ Substance use disorders are probably underreported.^30^ However, a diagnosis that is recorded is expected to be valid. The diagnosis groups were different in many of the characteristics, which are related to the employment rate: persons with schizophrenia were older, had lower education, and were less often married than persons with bipolar disorder or the general population. Compared to the general population, people with bipolar disorder were older and were less often married or cohabiting, and women were overrepresented.

The association of demographic and clinical factors as well as the local employment rate was mostly in the same direction among people with schizophrenia and bipolar disorder. However, factors possibly describing a milder course of illness or better social functioning (being married, later age of onset and higher level of education) were related to a higher employment rate, especially among people with schizophrenia, whereas among people with bipolar disorder, the effect of substance use disorders was pronounced. Men with bipolar disorder were more likely to be working than women with this disorder. The difference is congruent with the results in some studies.^31^, ^32^ The sex difference may partly stem from differences in the symptomatology: Women with bipolar disorder spend more time depressed and have more often a rapid‐cycling illness course than men.^31^, ^32^ Both depressive symptoms as well as a rapid‐cycling course have been related to a lower probability of working.^14^, ^29^, ^33^ However, the difference may also reflect gender differences in the employment rate in the general population.^25^ The result of no sex difference in employment rate among people with schizophrenia was expected.^10^

Besides patient‐related characteristics, the environment and society affect the patients’ possibilities of being employed. In the present study as well as in the study by Marwaha et al^10^ focusing on schizophrenia, the local employment rate was associated with the probability of employment. For example, the employment rate among people with a bipolar disorder in the year 2013 was 27% in the municipalities with the lowest employment rate quartile and 37% in the municipalities with the highest employment rate quartile.

Changes over time were noted especially in people with bipolar disorder. The most striking change was a decrease in the disability pension followed by an increase in the proportion of the unemployed, which may be explained by the tightening of eligibility requirements for sickness absence and disability pensions in the year 2008. Similar but smaller changes were noticed in the general population as well as in people with schizophrenia.

Diagnosing bipolar disorders has increased during the study years, and the diagnosis is being received earlier. Carlborg et al^11^ discuss that the increase may result from improved recognition of bipolar disorder or a change in the diagnostic threshold.

In the present study, the employment rate—when defined as the main activity of the year—was low compared to the previous studies.^7^, ^8^, ^9^, ^10^, ^34^ The explanation for the low employment rate may be that the individuals whose main activity of the year was other than work (e.g., a disability pension) were put into categories other than work. Accordingly, other definitions of employment examined in the present study yielded a higher employment rate. In previous studies, where employment has been defined, the definition has varied in relation to whether salary level (e.g. earning a minimum wage) or duration of the employment (e.g. working full‐time or at least some specified time in a week) is used, which employment type is considered (competitive employment or any employment) and whether students or stay‐at‐home parents have been included among the employed.^18^, ^34^, ^35^ Because the definition of employment varies besides variation in sampling frame as well as between societies, it is difficult to compare the employment rate in different studies. The current way of defining employment in terms of the main activity of the year is based on previous articles where the method has enabled an explicit presentation of the complex dimensions of the main activity and source of income.^26^ In addition, the use of earnings as a continuous variable was hindered by the fact that people with schizophrenia or bipolar disorder often do not have any income.^36^ Dichotomizing the outcome variable enabled us to run the appropriate analyses. Future studies should address employment in more detail, for example, as salary level, profession, or quality of employment.

The strengths of the study include the use of a nationwide database where all people living in the country are recorded. In addition, we had a long follow‐up during which there was no attrition except that due to mortality or emigration. A limitation of the present study is that only variables that can be found in the registers could be used, and therefore, information on symptom severity was lacking. Also, due to limitations in the register data, we were not able to differentiate bipolar I and bipolar II disorder. The definition of incidence differed in the cohorts of patients with schizophrenia and bipolar disorder due to the availability of data in the registers and the specificities of pharmacological treatment for these disorders. More concretely, due to the higher variability and lower specificity of medication used for treating bipolar disorder as opposed to schizophrenia, data on medication could not be used in the selection of individuals with bipolar disorder to the incidence group. Still, the onset of bipolar disorder seems to be well‐defined given the declining employment rates and increasing sick leave and disability pension rates around the time of the first register diagnosis.

In conclusion, employment is relatively rare in people with schizophrenia, and most of them are on a disability pension. Employment is more common among people with bipolar disorder. Although both disorders cause heavy impairments in work functioning, many interventions—such as Individual Placement and Support—have been proven effective in increasing labor market participation.^37^, ^38^ However, the interventions are not often used.^39^ Evidence‐based support for gaining and maintaining employment should be publicly available and easily accessible for people with impaired work ability. The importance of work for the quality of life in people with severe mental disorder^14^, ^18^ should be acknowledged in the treatment system, and patients should be actively referred to vocational rehabilitation, especially early in the course of the illness. In addition, work should be used as an important outcome when studying treatment or rehabilitation.

## CONFLICT OF INTEREST

Jari Tiihonen, Heidi Taipale, Ellenor Mittendorfer‐Rutz, and Antti Tanskanen have participated in research projects funded by grants from Janssen‐Cilag and Eli Lilly to their employing institution. Heidi Taipale reports personal fees from Janssen‐Cilag. Jari Tiihonen reports personal fees from the Finnish Medicines Agency (Fimea), European Medicines Agency (EMA), Eli Lilly, Janssen‐Cilag, Lundbeck, and Otsuka. He a member of the advisory board for Lundbeck and has received grants from the Stanley Foundation and the Sigrid Jusélius Foundation.

## Data Availability

We are not allowed to make the data used in this study available to the public.
