# Supplementary material for: Employment among people with schizophrenia or bipolar disorder: A population‐based study using nationwide registers
Source: Acta Psychiatr Scand. 2020 Nov 24;143(1):61–71. doi: 10.1111/acps.13254 (PMC7839734; doi:10.1111/acps.13254)
Supplement: Supplementary file 1 [file ACPS-143-61-s001.pdf]

Supplementary Table 1. The construction and values of the study variables.

	Construction	Values	Register
Diagnosis	Schizophrenia (International Classification of Diseases 10th Revision, ICD-10 code F20) or bipolar disorder diagnosis (ICD-10-codes F30-F31) during years 2006–2013.	Schizophrenia / Bipolar disorder	The National Patient Register and Micro-Data for Analysis of the Social Insurance System (MiDAS) register
Main activity of the year	Statistics Sweden categorized people as employed when they received income from work above a limit, which approximates whether one has been working at least one hour in a week. People who were unemployed, on parental leave, on sick leave (as gross days), on disability pension (as gross days) or on old-age pension over half of the year, who received more than half of their disposable income from student benefits or from social assistance and the remaining individuals (categorized as “other”) were categorized as such.	Employed / Student / Parental leave / Unemployed / Social Assistance / Other / Sick-leave / Disability Pension / Old-age Pension	Longitudinal Integration Database for Health Insurance and Labour Market Studies (LISA)
Employment	Employment was defined based on the main activity of the year (see preceding row).	Employed / Not Employed	LISA
Age	Age reached 31/12 each year.	For the Tables categorized as 18–24 / 25–34 / 35–44 / 45–54 / 55–64. In the GEE analysis, age was used as a continuous variable.	LISA
Gender		Woman / Man	LISA
Education	The highest level of education reached was put into three categories: Low (compulsory school, 9 years of education or less), Middle (upper secondary education, 10–12 years of	Low / Middle / High	LISA

	education) and High (college or university).		
Marital Status	For marital status, people were divided into the categories married or cohabiting and others.	Married or Cohabiting / Other	LISA
Immigration Status	Immigration status was defined as whether the person was born in Sweden.	Born in Sweden / Born in another country	LISA
Municipality Employment Rate	Municipality employment rate was defined as the percentage of people in each municipality whose main activity of the year was defined as employed.	Lowest 25% of municipalities / Middle / Highest 25% of municipalities. In the GEE analysis, used as a continuous variable.	LISA
Age at First Diagnosis	The age of first diagnosis of F20–F29 in the schizophrenia cohort or F30–F31 in the bipolar disorder cohort as the main diagnosis	< 25 years / 25–34 years / > 35 years	The National Patient Register and the MiDAS register
Substance Use Disorder Diagnosis	Register diagnosis of ICD-10 codes F10–F19 since the year 2001 and until the year in question (2006–2013)	Yes / No	The National Patient Register and the MiDAS register
Psychiatric Hospitalizations	Hospitalizations with a main diagnosis of F01–F99 starting from five years before the beginning of the follow-up (from the year 2001) until the year in question	No hospital care / 1–2 hospitalizations / 3–5 hospitalizations / > 6 hospitalizations	The National Patient Register
